# Morphological data of the genus *Lejeunea* (Marchantiophyta: Lejeuneaceae) in the Malesian region

**DOI:** 10.1016/j.dib.2019.104958

**Published:** 2019-12-07

**Authors:** Gaik Ee Lee

**Affiliations:** a Faculty of Science and Marine Environment, Universiti Malaysia Terengganu, 21030, Kuala Nerus, Terengganu, Malaysia; b Institute of Tropical Biodiversity and Sustainable Development, Universiti Malaysia Terengganu, 21030, Kuala Nerus, Terengganu, Malaysia

**Keywords:** Morphology, Leafy liverwort, *Lejeunea*, Lejeuneaceae

## Abstract

The morphological data of Malesian *Lejeunea* is described in detail based on examination of about 600 fresh specimens and 1500 herbarium specimens of *Lejeunea* from other herbaria. Scanning electron microscopy (SEM) images and photographs illustrating the characters of *Lejeunea* are presented.

**Table:** Specifications Table

Subject	Botany, Taxonomy
Specific subject area	Morphology
Type of data	Table, figure
How data were acquired	Plant field collection, loaned herbarium specimens from other herbaria, examination of specimens by using light microscopy and scanning electron microscopy (SEM)
Data format	Raw and Analyzed
Experimental factors	Observation of oil bodies within 24 hours after collecting of the material.
Experimental features	Morphological study was based on the examination of approximately 600 fresh specimens and about 1500 herbarium specimens (including type materials) deposited in other herbaria e.g., the Conservatoire et Jardin botaniques de la Ville de Genève, Switzerland (G), Herbarium Haussknecht, Germany (JE), and the Hattori Botanical laboratory, Japan (NICH).
Data source location	There were 30 sampling localities, of which several are located in each State of Malaysia and Indonesia.
Data accessibility	All raw data are available within this article
Related research article	G.E. Lee, A Systematic revision of the genus *Lejeunea* Lib. (Marchantiophyta: Lejeuneaceae) in Malaysia, Cryptogamie, Bryologie, 34 (2013) 381–484. https://doi.org/10.7872/cryb.v34.iss4.2013.381

**Table:** 

**Value of the Data** • The morphological data of Malesian *Lejeunea* could provide a basis for future works in this genus globally as the world-wide monograph or revision of Asian region does not exist. In addition, the data could enhance the knowledge of the genus due to its complex taxonomic history and the controversial relationships among its allies. • The data is useful for morphological identification as well as the study of character state evolution of *Lejeunea.* • The data e.g., the oil bodies could serve as a reference or baseline information for other future applied research such as phytochemistry and biological activity of secondary metabolites.

## 1 Data

The morphological characteristics of the genus include:

1) Plants habit, texture and colour

**Fig. 1:**
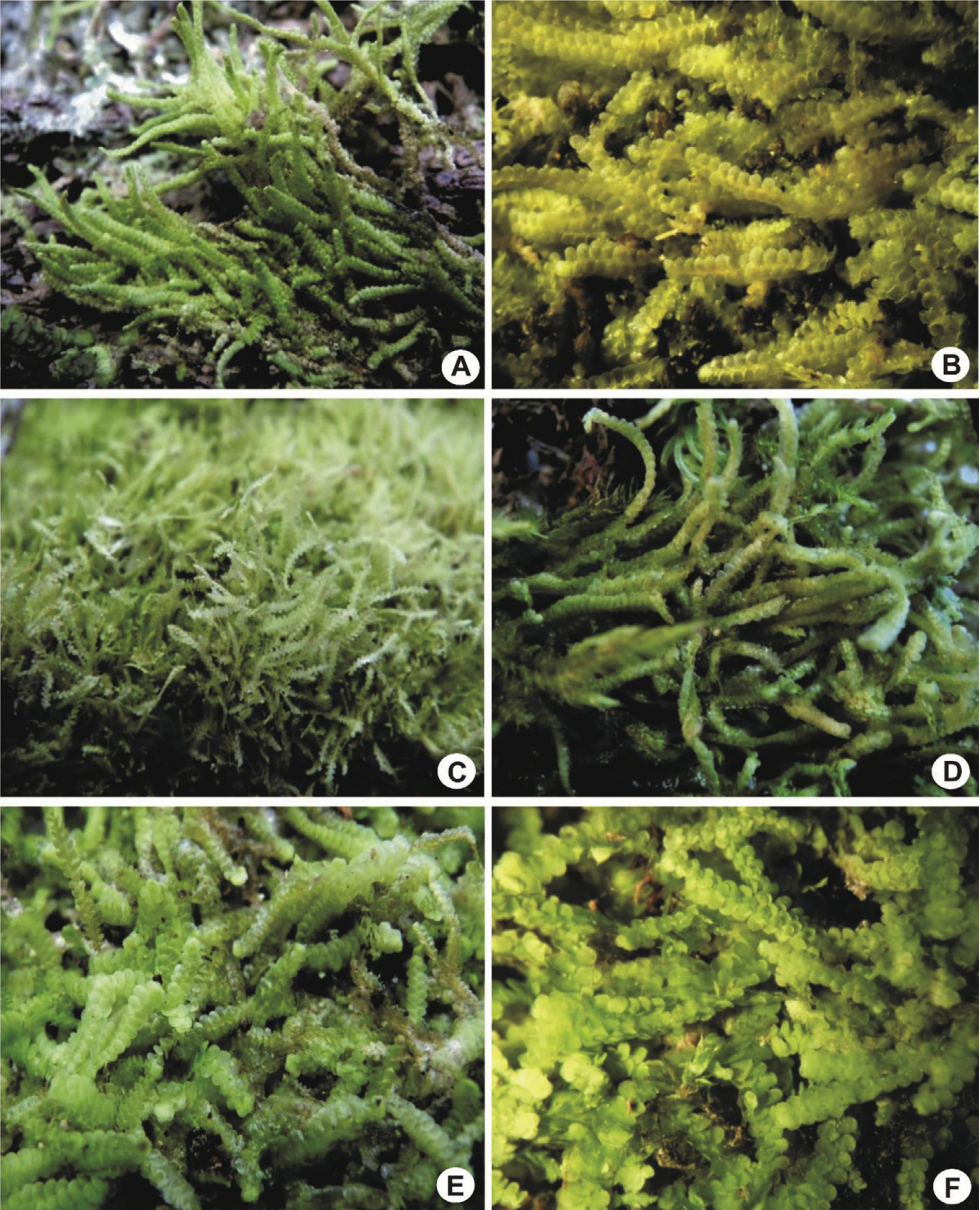
Habit of *Lejeunea*. A: *L. discreta*; B: *L. micholitzii*; C: *L. tuberculosa*; D: *L. kinabalensis*; E: *L. mimula*; F: *L. sordida*.

Species of *Lejeunea* are usually found growing in loose mats and patches or occasionally tightly appressed to the substrates. In some species, the plants are found growing in pendulous festoons or hanging down loosely from tree branches e.g., *L. lumbricoides*. The plants are small, delicate or medium sized (0.5–1.5 mm wide with the leaves) to somewhat robust (up to 2.0 mm) and usually are soft-textured. When fresh, the plants in general are yellowish or whitish (somewhat glossy) to pure or pale green in colour (Fig. 1).

2) Stems and branching

**Fig. 2:**
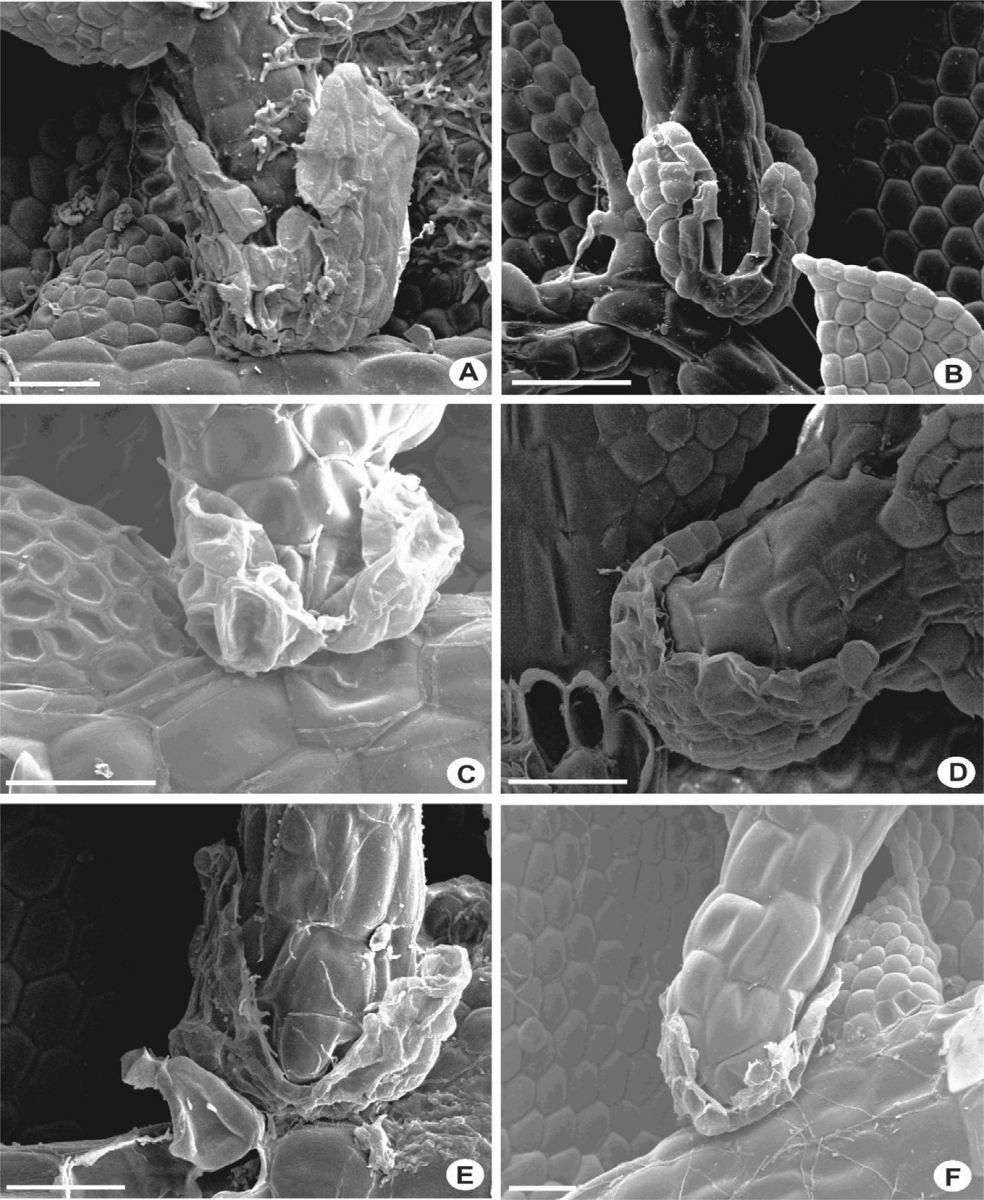
Portion of branches, showing three-lobed basal collar. A: *L. albescens*; B: *L. micholitzii*; C: *L. umbilicata*; D: *L. flava*; E: *L. microloba*; F: *L. mimula*. Scale bar A–F: 50 μm.

Stems with hyalodermis and ventral merophytes 2 cells wide. Epidermal cells larger than medullary cells, usually 7 cells except in *L. tjibodensis*, 9 [1]. The branches are exclusively *Lejeunea*-type and always with 3-lobed of basal collar of which two lobes are situated at the ventral side and one lobe on the dorsal side. Most species of *Lejeunea* have small collar, 0.07–0.1 mm long (Fig. 2C–E), however, some larger plants e.g., *L. albescens* and *L. lumbricoides* have larger collar, 0.15–0.25 mm long (Fig. 2A, B).

3) Underleaves

**Fig. 3:**
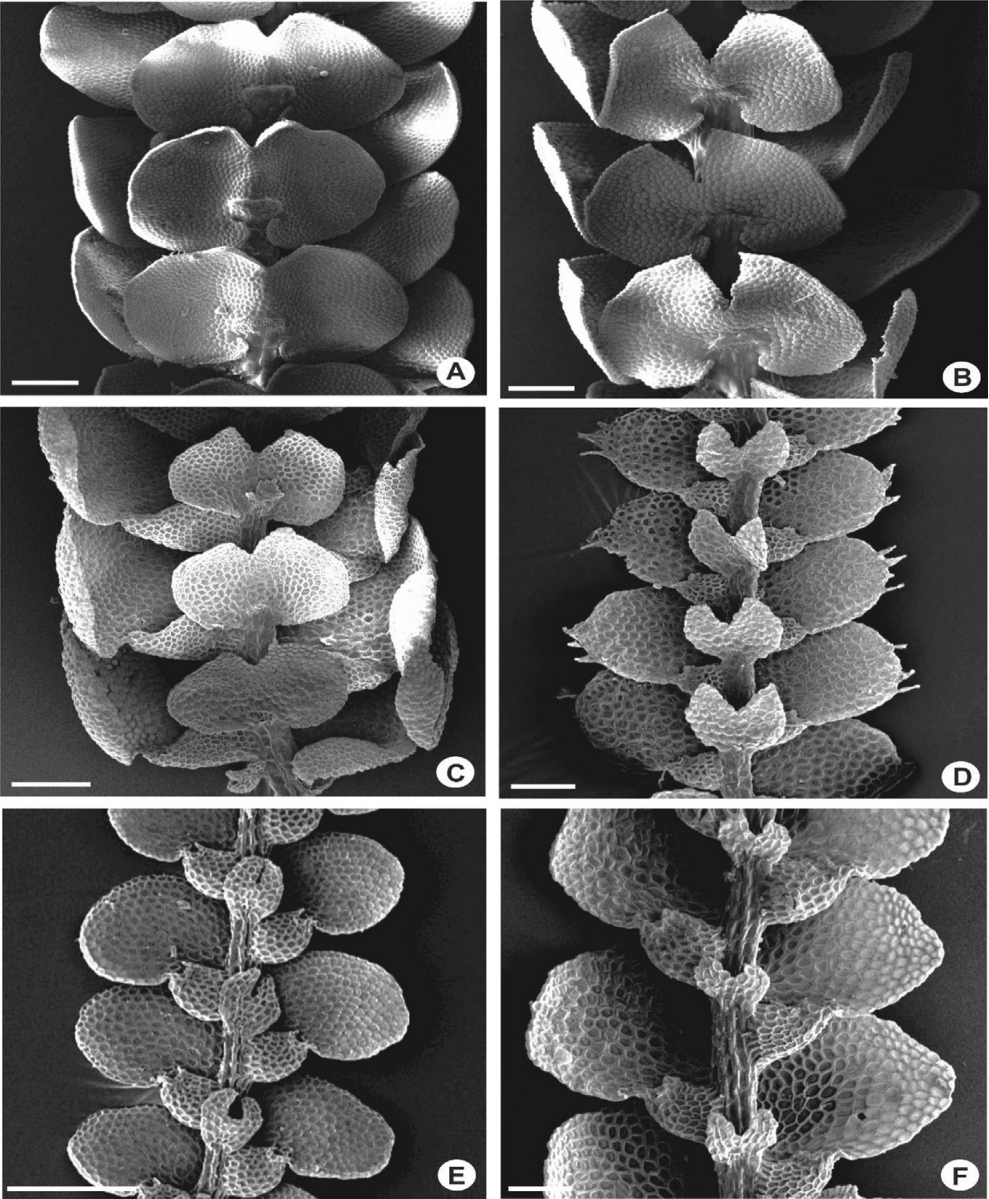
Portion of shoots, showing underleaves. A: *L. albescens*; B: *L. kinabalensis*; C: *L. pectinella*; D: *L. patriciae*; E: *L. tuberculosa*; F: *L. pulchriflora*. Scale bar A–F: 200 μm.

The underleaves vary from suborbicular to reniform, bilobed in all of the taxa except in *L. mimula* and *L. leratii*, imbricate to distant, 2–7 times wider than the stem, 0.1–1.0 mm long and 0.1–1.0 mm wide. Underleaves bilobed to 1/5–4/5 of the underleaf length. In several species e.g., *L. albescens, L. compacta, L. contracta, L. dipterota, L. flava*, *L. kinabalensis, L. koordersii, L. microloba, L. mimula, L. sordida* and *L. tjibodensis* the underleaves are large to the extent of covering the whole leaf lobules (Fig. 3A, B), whereas some are only covering 1/2–3/4 of the leaf lobules e.g., *L. javanensis*, *L. pectinella* and *L. patriciae* (Fig. 3C, D). Small underleaves are seen in *L. anisophylla*, *L. cocoes*, *L. eifrigii, L. exilis, L. patersonii* and *L. pulchriflora* (Fig. 3F). The lobes are 4–20 cells wide, lanceolate to shallowly triangular and usually oblique. The underleaf margins are entire to strongly crenulate with projecting cells. The underleaf bases are cuneate, almost straight to shallowly curved, or cordate to more or less auriculate.

**Fig. 4:**
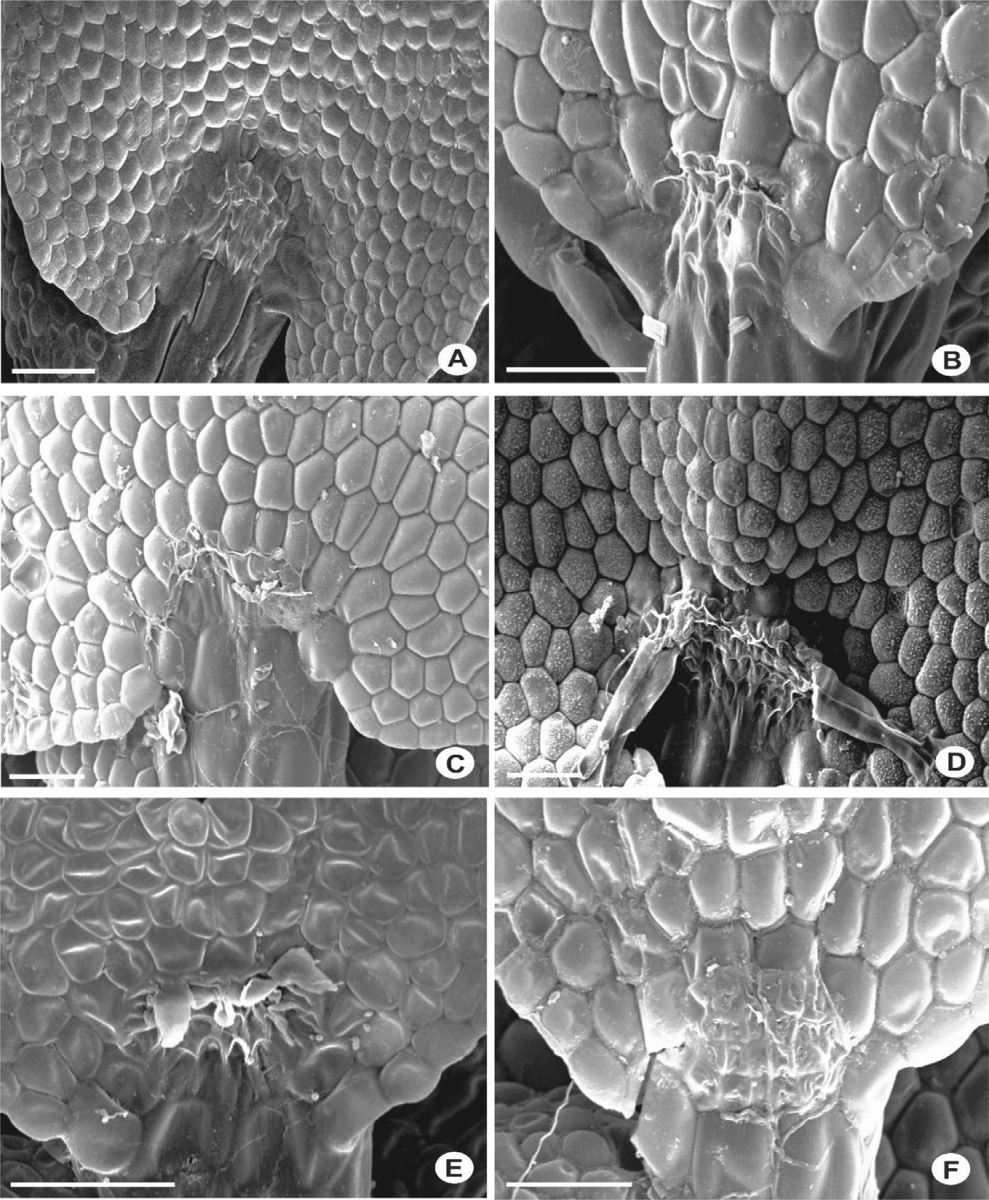
The base of underleaves, showing rhizoid initial cells. A: L. flava; B: *L. micholitzii*; C: *L. mimula*; D: *L. kinabalensis*; E: *L. alata*; F: *L. umbilicata*. Scale bar A–F: 50 μm.

**Fig. 5:**
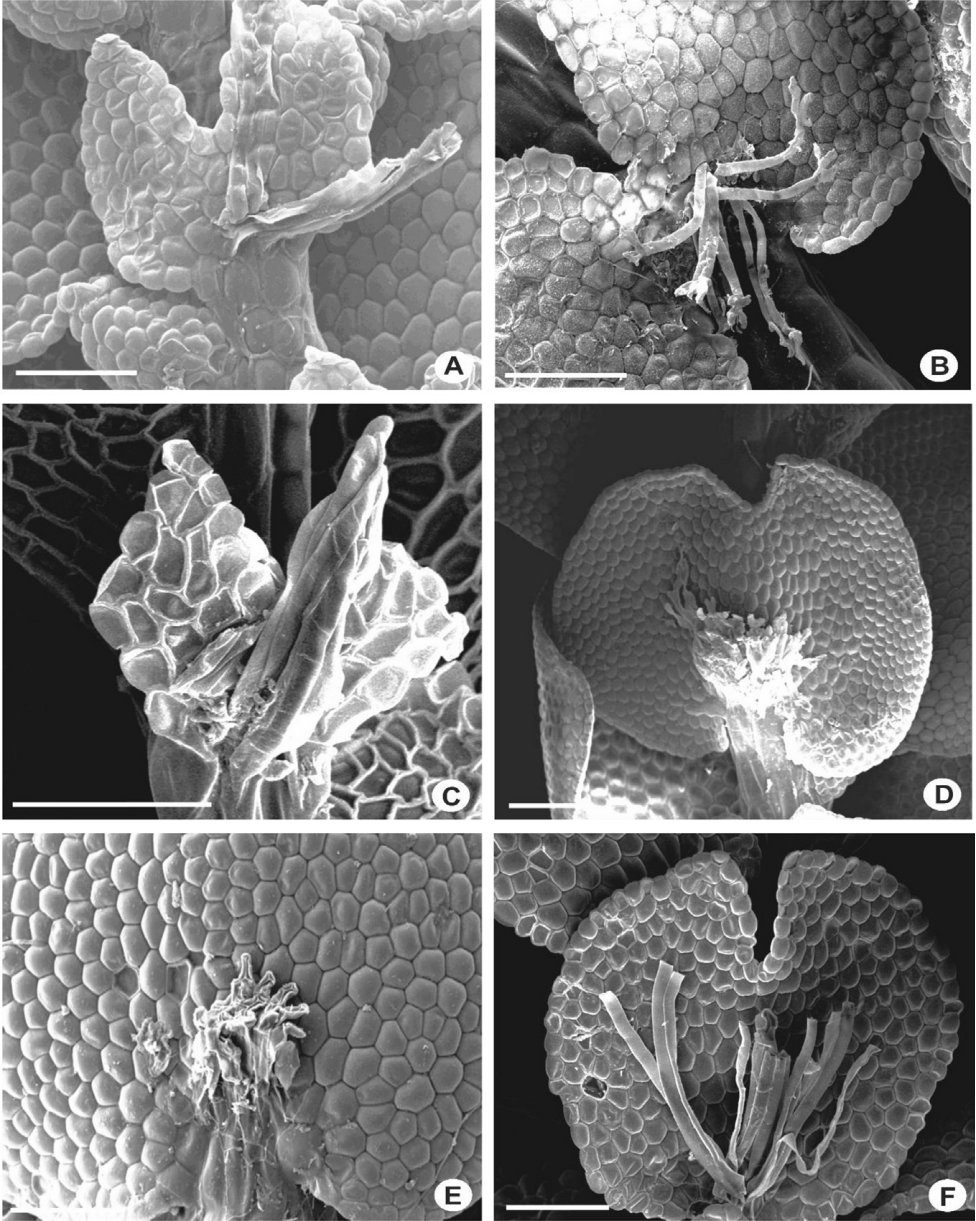
The base of underleaves, showing primary rhizoid disc. A: *L. umbilicata*; B: *L. kinabalensis*; C: *L. pulchriflora*; D: *L. albescens*; E: *L. mimula*; F: *L pectinella*. Scale bar A–F: 100 μm.

**Fig. 6:**
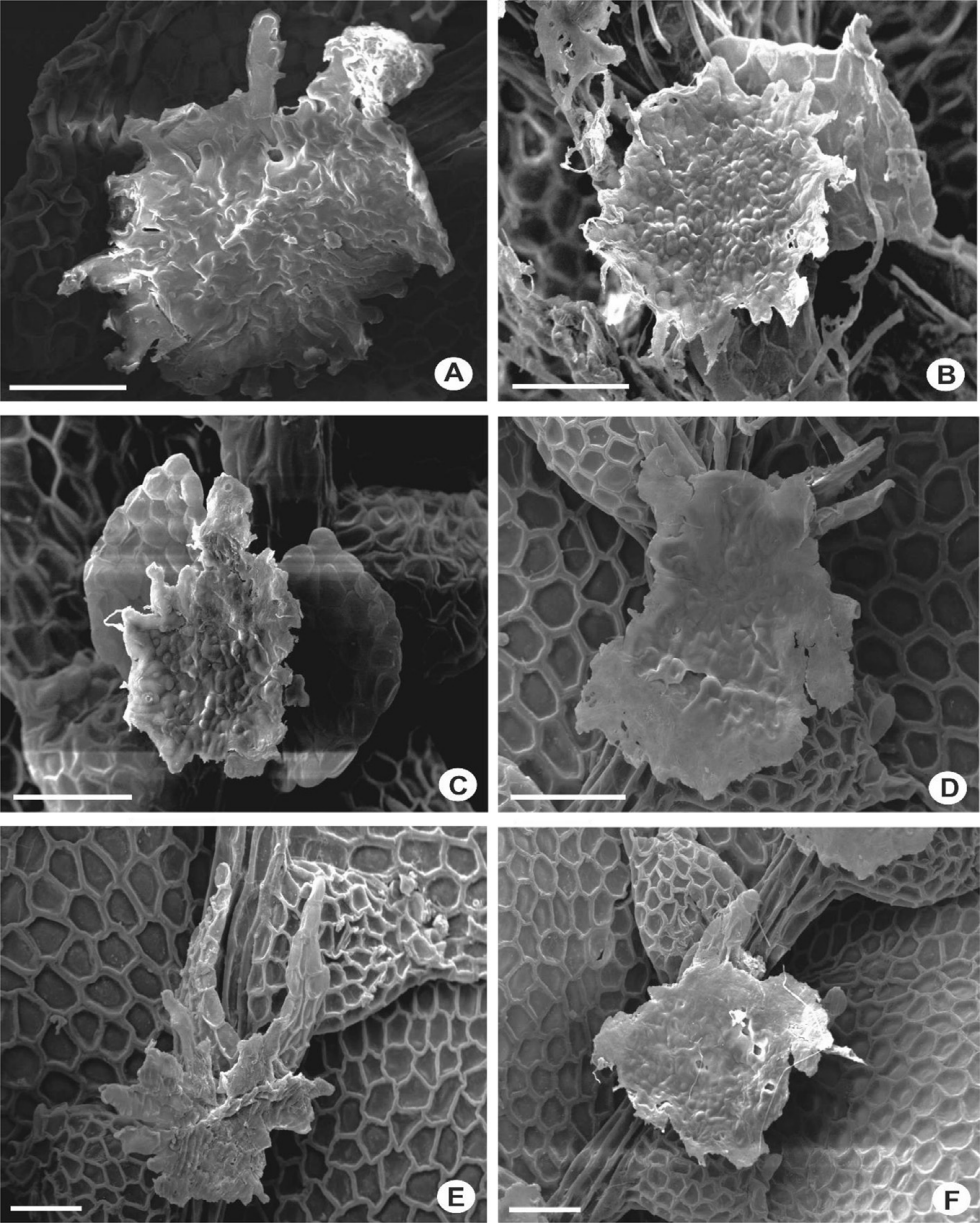
The base of underleaves, showing secondary rhizoid disc. A–C: *L. dimorpha*; D–F: *L. papilionacea*. Scale bar A–F: 50 μm.

**Fig. 7:**
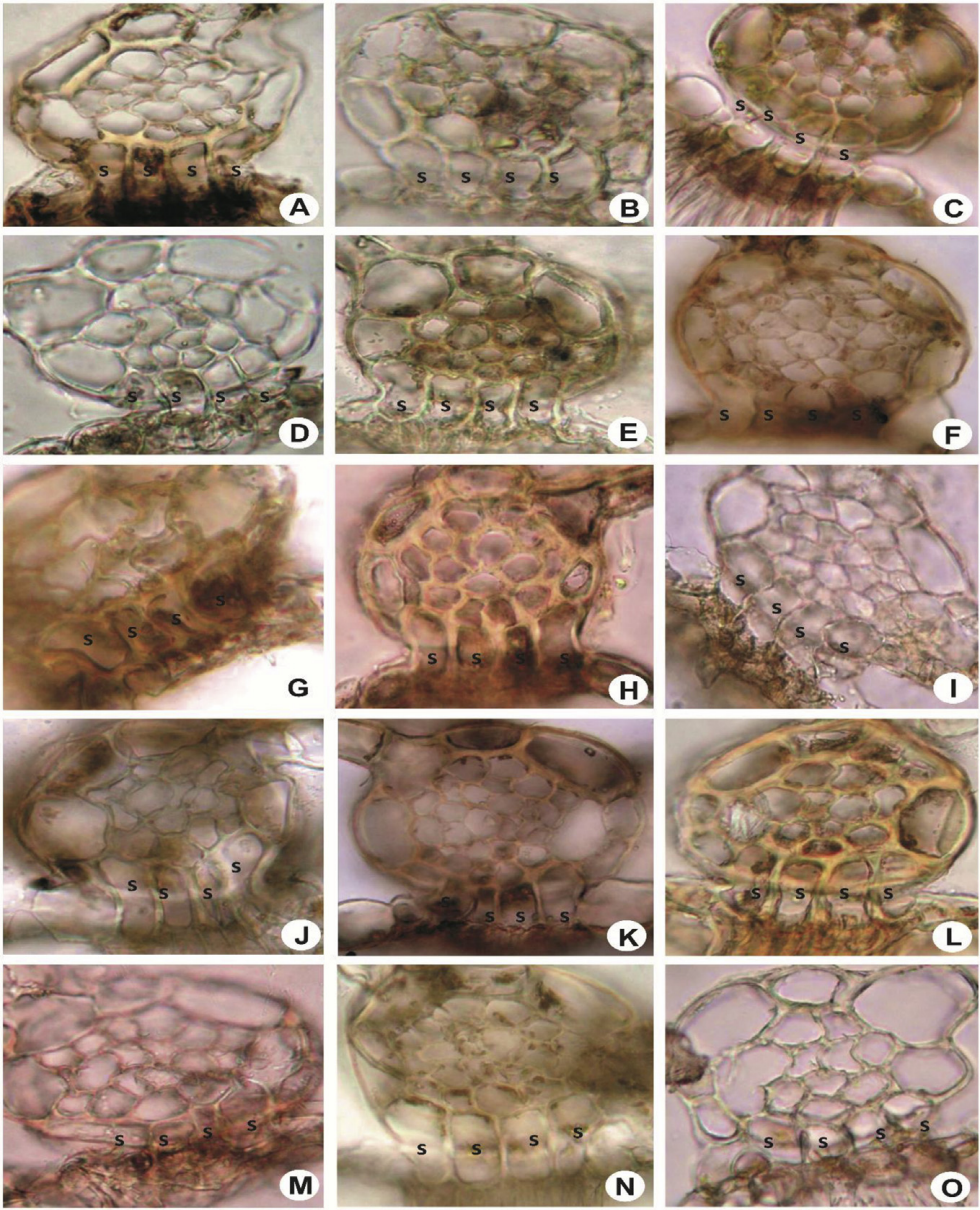
Stem in cross-section, showing superior central cells (s). A: *L. pectinella*; B: *L. stephaniana*; C: *L. umbilicata*; D: *L. tuberculosa*; E: *L. gradsteinii*; F: *L. kinabalensis*; G: *L. contracta*; H: *L. flava*; I: *L. mimula*; J: *L. dimorpha*; K: *L. mizutanii*; L: *L. utriculata*; M: *L. microloba*; N: *L. alata*; O: *L. patersonii*.

**Fig. 8:**
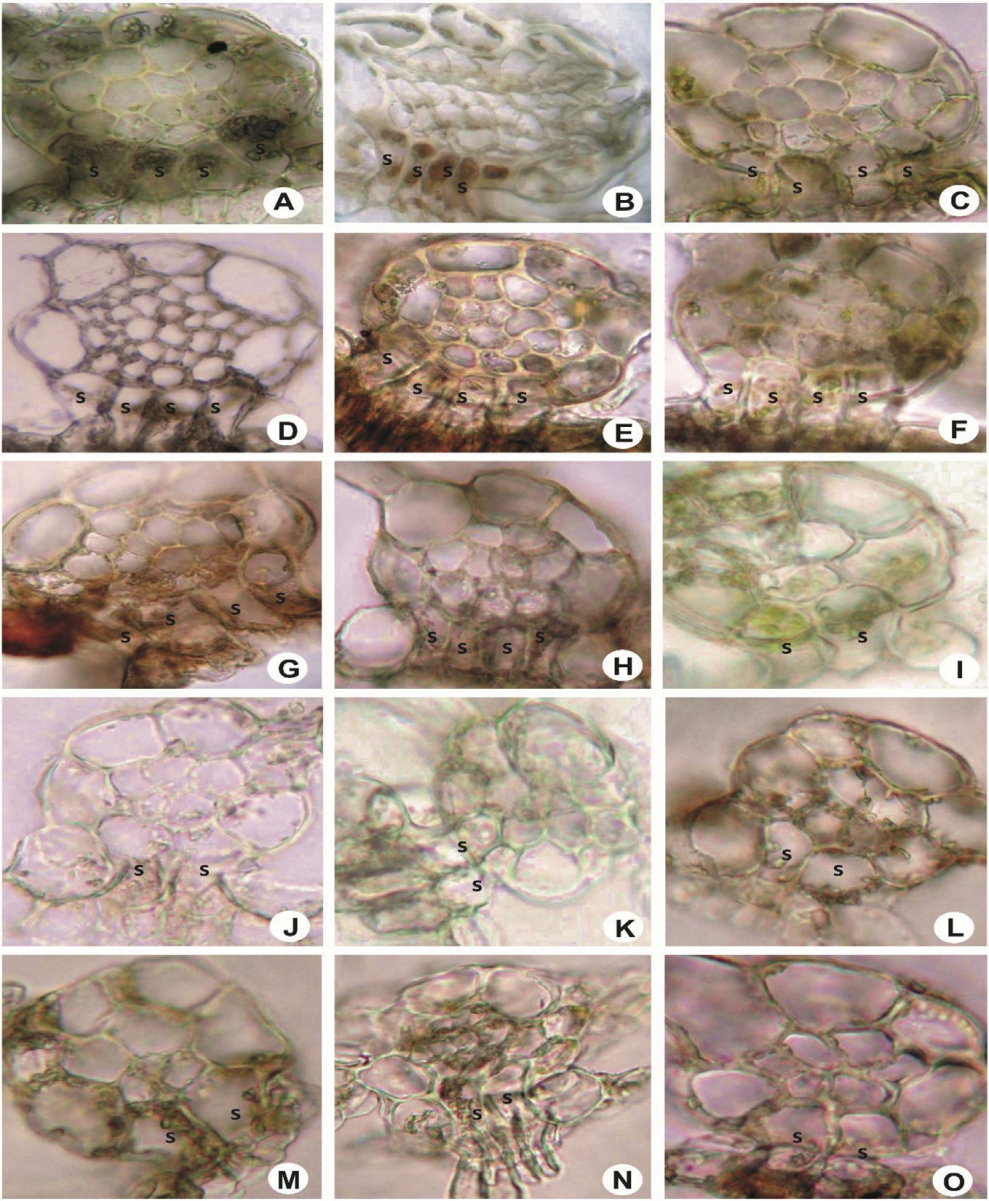
Stem in cross-section, showing superior central cells (s). A: *L. sordida*; B: *L. lumbricoides*; C: *L. micholitzii*; D: *L. eifrigii*; E: *L. albescens*; F: *L. dipterota*; G: *L. compacta*; H: *L. discreta*; I: *L. cocoes*; J: *L. patriciae*; K: *L. exilis*; L: *L. pulchriflora*; M: *Lejeunea* sp.; N: *L. papilionacea*; O: *L. anisophylla*.

**Fig. 9:**
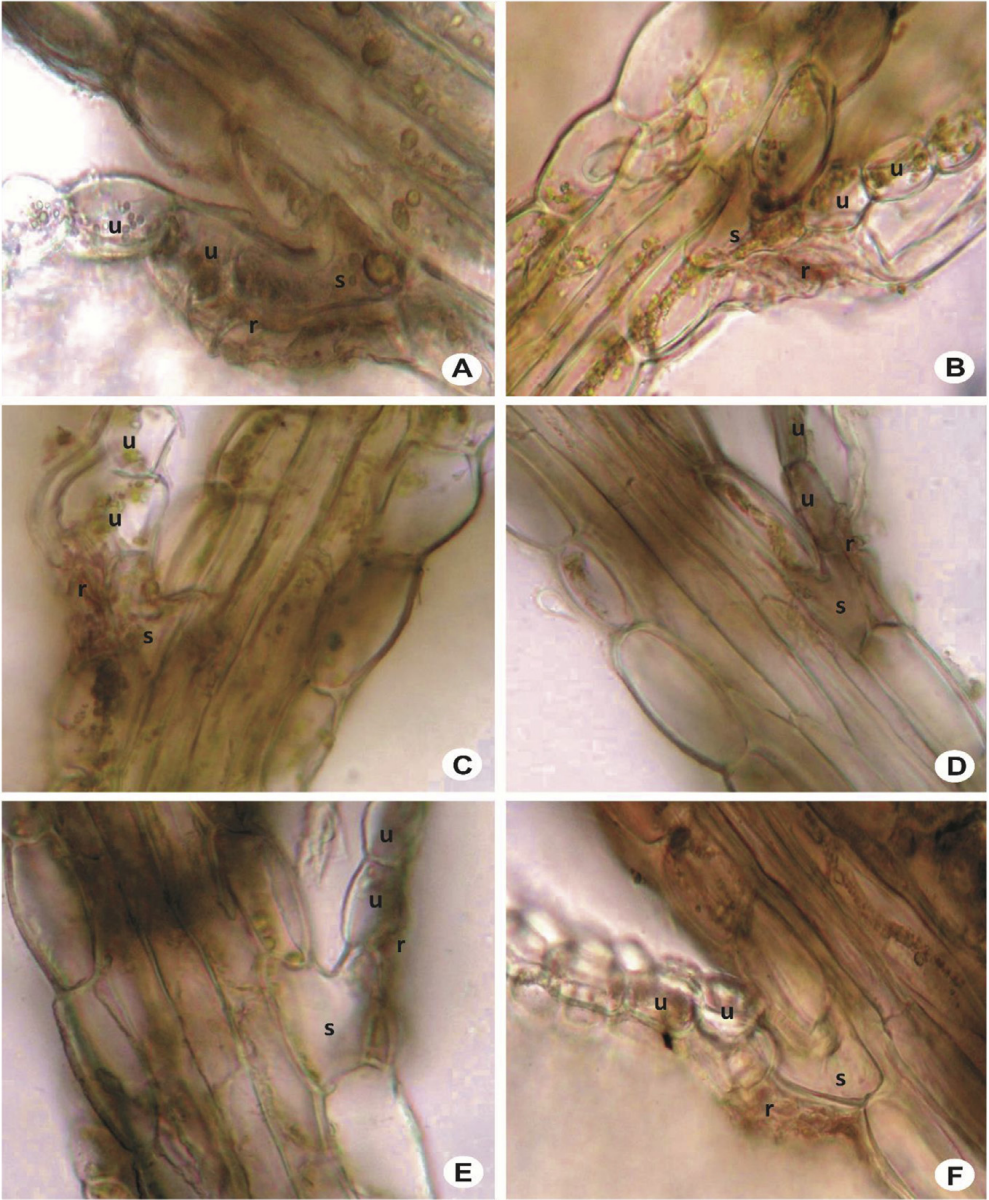
Stem in longitudinal section, showing superior central cell and bistratose underleaf base. A & B: *L. flava*; C & D: *L. umbilicata*; E & F: *L. tuberculosa*. s = superior central cell, u = underleaf cell, r = rhizoid.

The primary rhizoid disc or the rhizoid-initial area originated from a small group of bulging cells or rhizoid initial cells (Fig. 4) and forms a bundle of short unicellular rhizoids, which attach the plant to the substrate. Sometimes the apices of the rhizoids grow into a hand-shaped pattern (Fig. 5B). The secondary rhizoid disc consists of joined apices of the rhizoids protruding from the underleaf base and forms a conspicuous circular mat (Fig. 6). The underleaf bases are attached to the stem by superior central cells, inferior central cells and two large basal underleaf cells. In cross-sections, the superior central cells are usually elongated or rectangular in shape as compared to adjacent cells (Fig. 7, Fig. 8) while in longitudinal sections, the superior central cell is large and U-shaped (Fig. 9). See Ref. [2] for further description on the anatomy of underleaf bases. The primary rhizoid disc consists of 10–50 thin-walled rhizoid initial cells (Fig. 4). They are somewhat of the same size or smaller than the surrounding underleaf cells. The primary rhizoid discs are usually present, mostly hyaline to brownish, long filamentous, in loose fascicles, at the base of the underleaves (Fig. 5) whereas secondary rhizoidal disc usually lacking, sometimes present in *L. anisophylla, L. dimorpha* and *L. papilionacea* (Fig. 6). The cross-section of the stem shows two or four superior central cells (Fig. 7, Fig. 8). In longitudinal sections, the underleaf base of *Lejeunea* is always bistratose (Fig. 9).

4) Leaves

**Fig. 10:**
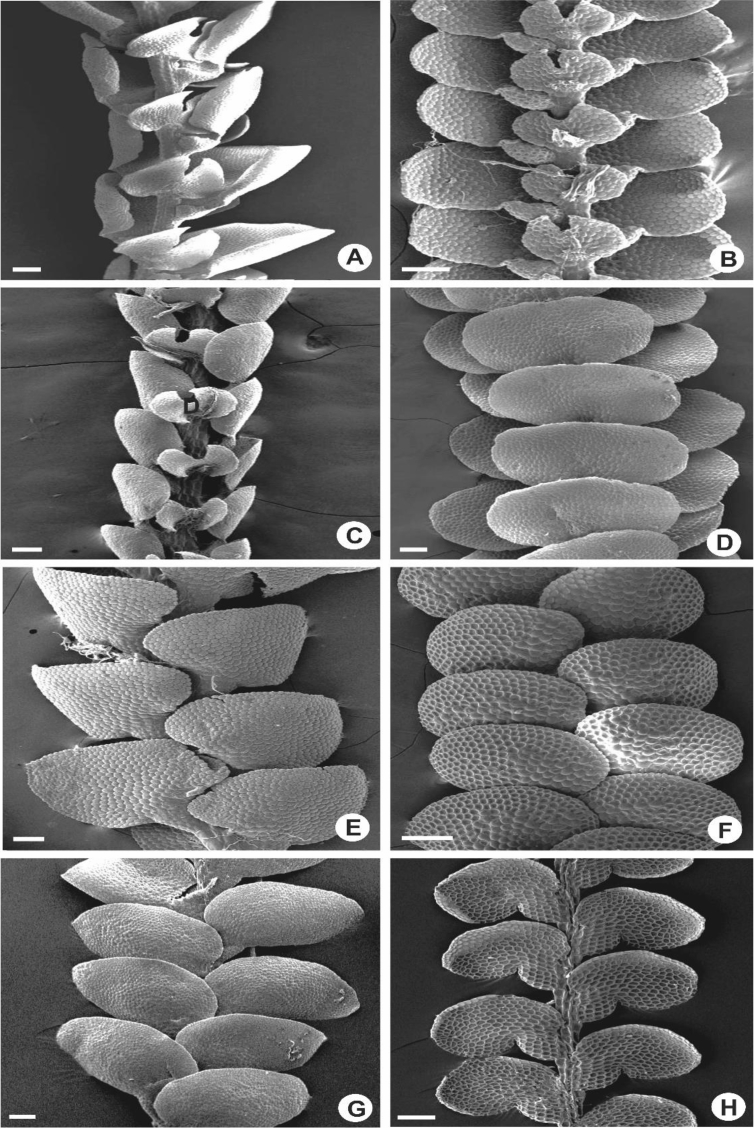
Portion of shoots (A–D: ventral view; E–H: dorsal view). A: *L. lumbricoides*; B,F: *L. umbilicata*; C: *L. stephaniana*; D: *L. mimula*; E: *L. kinabalensis*; G: *L. flava*; H: *L. tuberculosa*. Scale bar A–H: 100 μm.

**Fig. 11:**
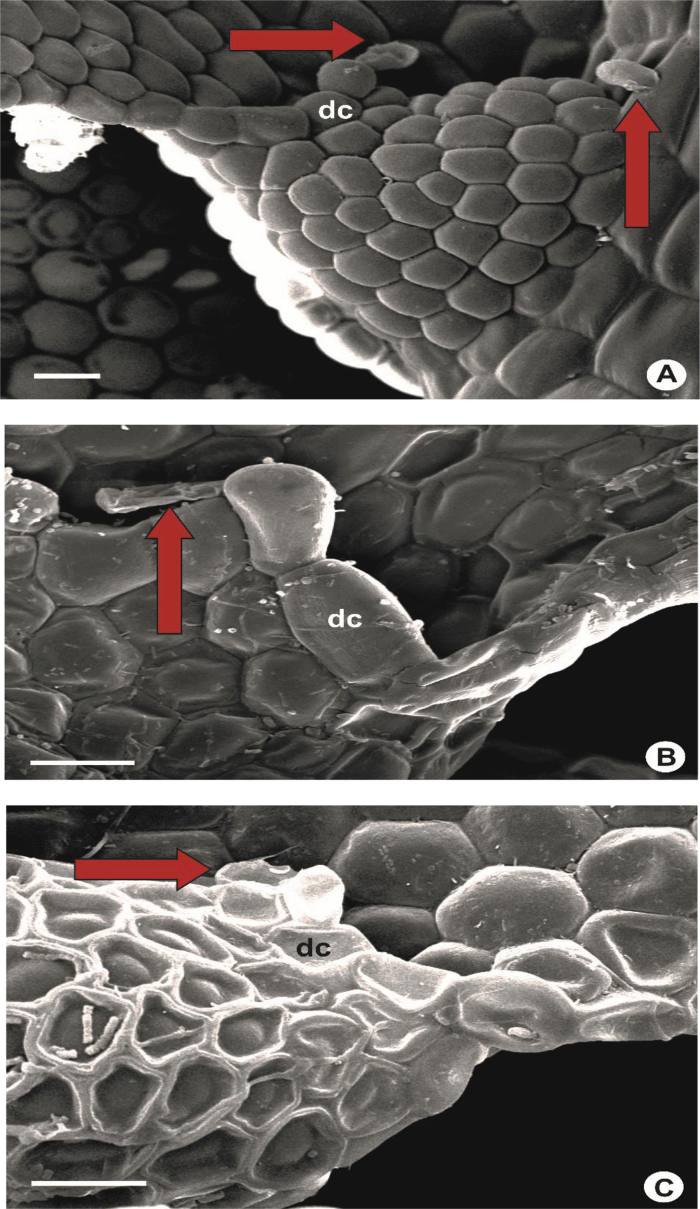
Portion of leaf lobules (ventral view), showing hyaline papillae in red arrow and disc cell (dc). A: *L. albescens*; B: *L. lumbricoides*; C: *L. patriciae*. Scale bar A–C: 200 μm.

The leaves of *Lejeunea* are complicate-bilobed in which the leaves are divided into a large dorsal lobe or leaf lobe and a smaller, inflated ventral lobe or leaf lobule (Fig. 10). The leaf lobe and lobule are attached to the stem along a J-shaped line of insertion, about 10–15 lobe cells long. The dorsal row of the leaf lobe is in a straight or almost straight longitudinal line and the line abruptly become slightly oblique at the apex (Fig. 10). The leaf lobule is attached to the stem by 5–9 lobule cells along a vertical line. The leaves of *Lejeunea* vary slightly in dry or moist condition. When dry, the leaves of robust plants are usually recurved, convex, and occasionally crispate and suberect whereas the minute leaves are usually flat in smaller plants and rarely convex. When moistened, the leaves are usually erect-spreading to spreading and plane, sometimes the leaves of robust plants are still recurved and weakly convex. The leaves have three hyaline papillae in which two are situated in the uppermost cell of insertion line of the leaf and one at the apex of leaf lobule (Fig. 11).

**Fig. 12:**
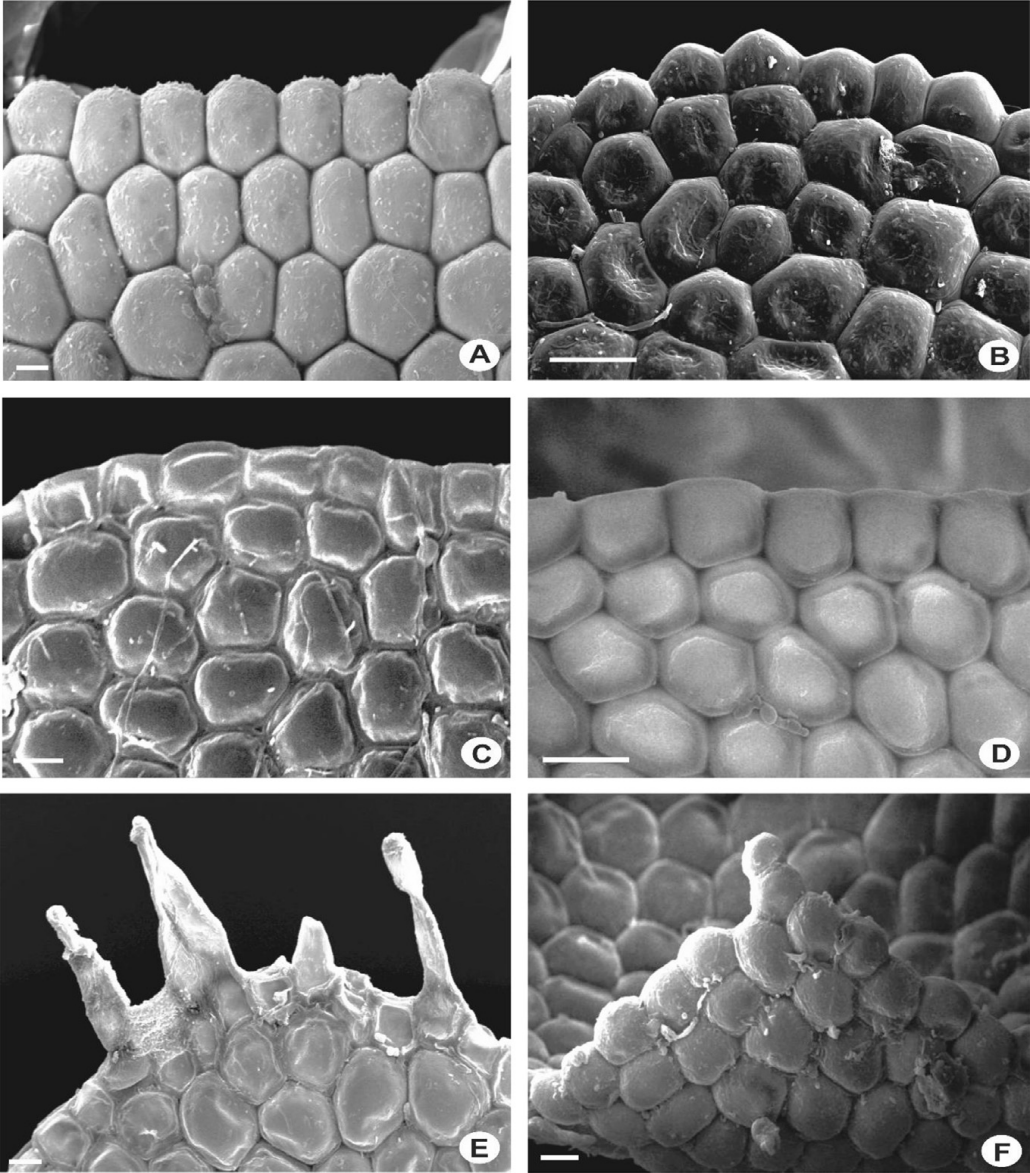
Margins of *Lejeunea*. A: *L. kinabalensis*; B: *L. patersonii*; C: *L. alata*; D: *L. flava*. E: Margin with rhizoids, *L. patriciae*; F: Margin crenulate with apiculate tip, *L. microloba*. Scale bar: 10 μm.

The leaf lobes are ovate, ovate-triangular, ovate-rectangular or ovate-orbicular in shape with the apex broadly to narrowly rounded in most of the taxa or sometimes apiculate such as in *L. eifrigii, L. koordersii, L. microloba, L. tjibodensis* and *L. thallophora*. Leaf margins are entire to strongly crenulate with projecting cells (Fig. 12). In *L. albescens* and *L. kinabalensis*, the leaf margins are conspicuously crenulate by the protruding cells of the margin (Fig. 12A). The development of marginal rhizoids at the leaf apex with 5–10 projecting cells is only recorded in *L. patriciae*. Leaf lobules are ovate, ovate-oblong or ovate-triangular, about 1/5–1/2 the length of the leaf lobe, consists of an inflated segment along the keel, a flattened or incurved segment along the free margin (the proximal base to the first tooth) and an apical margin (margin between first tooth and keel) [3]. Leaf lobules are usually situated at an angle of about 60°–90° to the stem and some are frequently reduced.

**Fig. 13:**
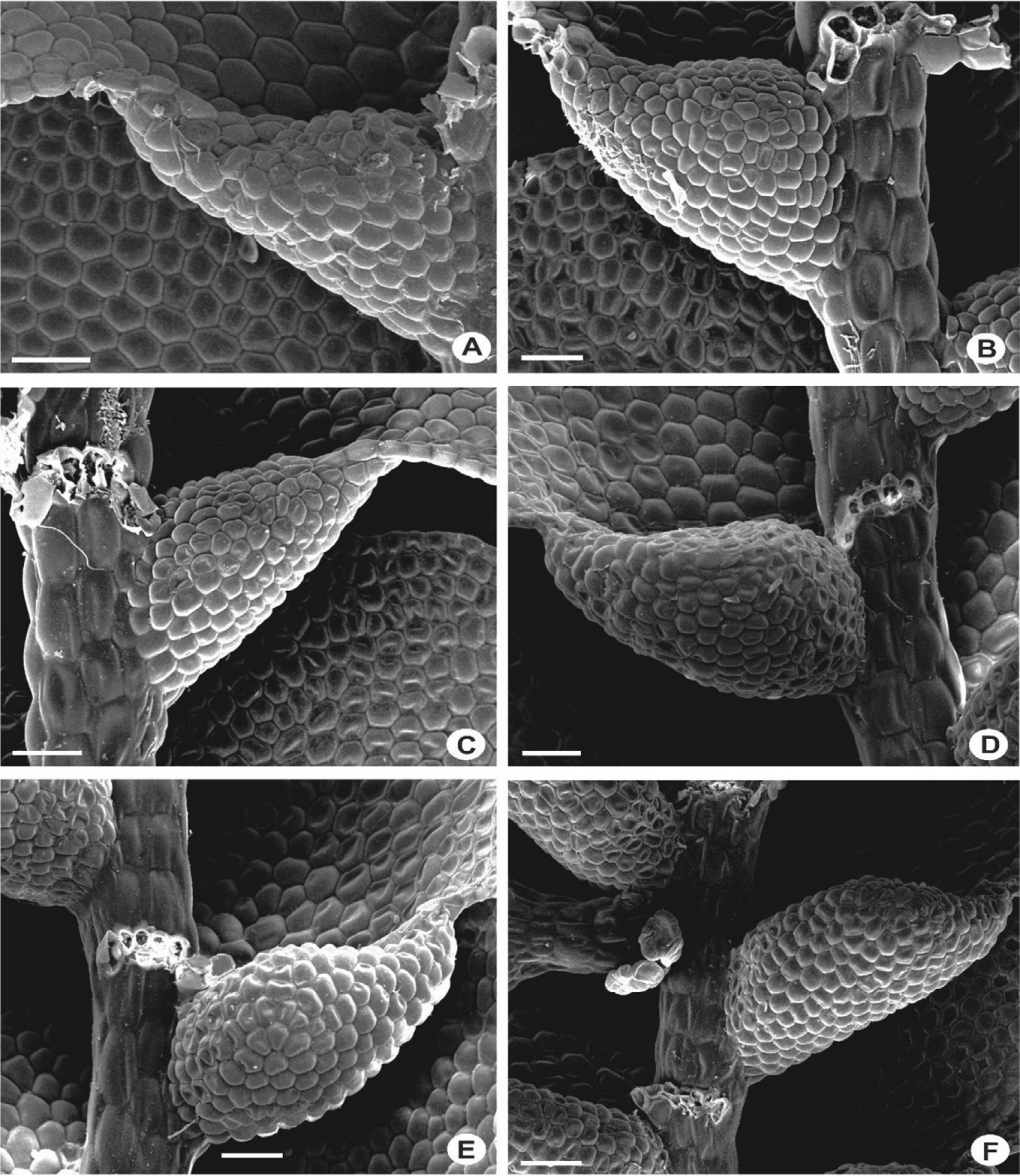
Leaf lobules, showing ovate-oblong and fully incurved free margins. A–C: *L. discreta*; D,E: *L. fleischeri*, F: *L. utriculata*. Scale bar A–F: 50 μm.

**Fig. 14:**
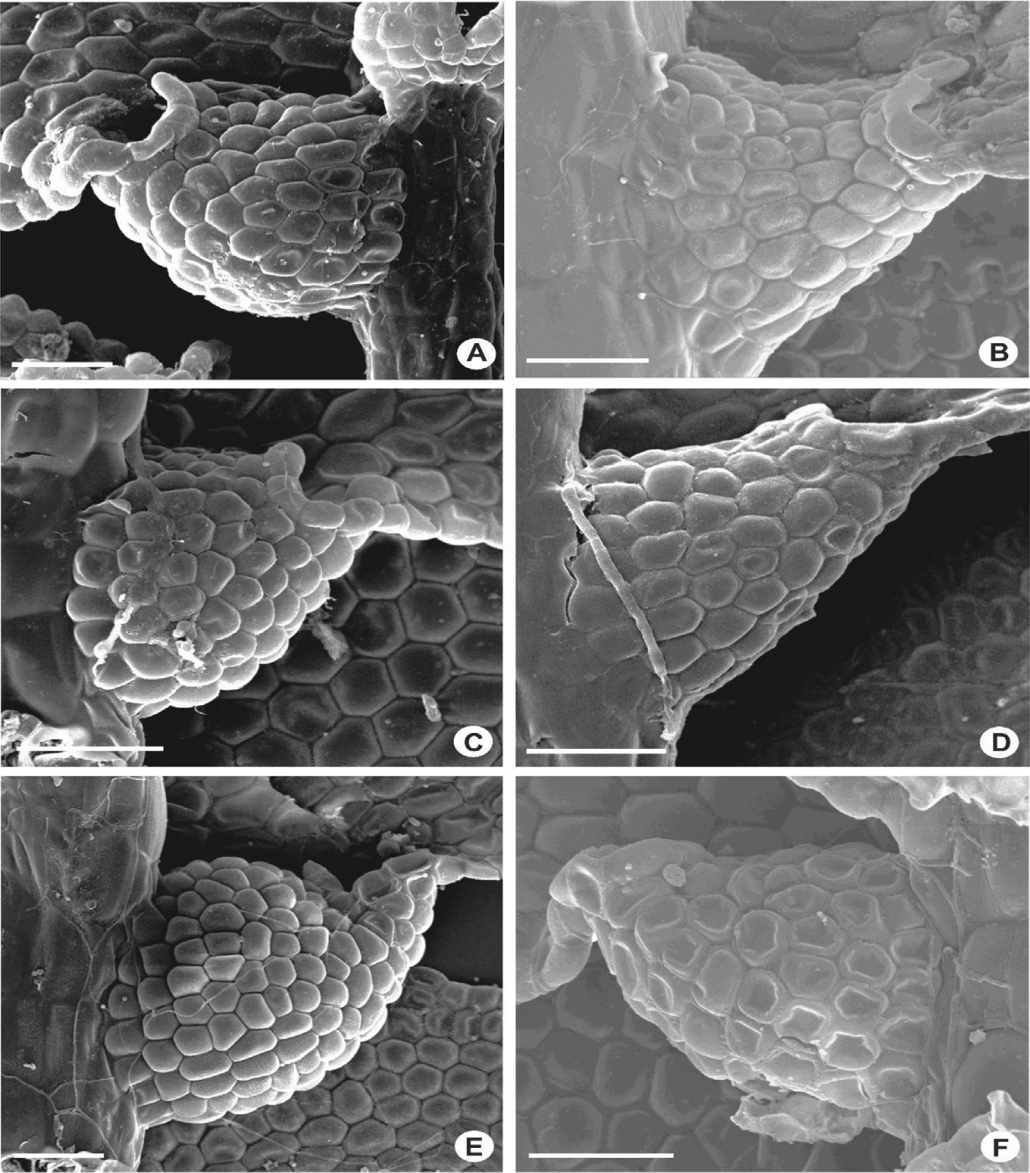
Leaf lobules, showing ovate or ovate-triangular shape and recurvation of free margins. A: *L. patersonii*; B: *L. kinabalensis*; C: *L. sordida*; D: *L. flava*; E: *L. dipterota*; F: *L. umbilicata*. Scale bar A–F: 50 μm.

The lobule apex is obliquely truncate to truncate. In *L. contracta, L. kinabalensis, L. koordersii* and *L. umbilicata*, the lobule apices are strongly curved or U-shaped when flattened. The free margins are usually either flat or partially or fully incurved (Fig. 13, Fig. 14). The partially incurved free margin is seen in some of the species e.g., *L. albescens, L. anisophylla, L. javanensis, L. micholitzii, L. mimula, L. papilionacea, L. sordida* and *L. wightii*, in which the free margin is incurved from the proximal base to 1/2–3/4 of the free margin length and subsequently abruptly flattened towards the apex. In this case, the apical margin which consists of first tooth, a rectangular disc cell and few adjacent cells of the lobe are clearly visible. In other species such as *L. dipterota*, *L. discreta, L. microloba, L. pectinella, L. splendida, L. stephaniana, L. umbilicata* and *L. utriculata,* the free margins are fully incurved, where the free margin together with the apical margin is incurved inside the lobule. In this situation, the whole apical margin cannot be observed in ventral view. However, the apical margin can readily be seen when the leaf lobule is detached from the stem. The free margin in most of the *Lejeunea* studied usually bears one-celled long tooth, however in *L. gradsteinii* and *L. splendida*, an obsolete and blunt second tooth is also seen and sometimes the first tooth is of 2-cells long. The first tooth is usually upward pointing, in which the position of the apical tooth is erect to suberect, mostly oblong in shape and the apex is obtuse and never acuminate.

The hyaline papilla is always situated at the proximal side of the first tooth (Fig. 11). In some species, a large rectangular cell (disc cell), very much larger than the first tooth, is found situated below the first tooth (Fig. 11) [4]. Species with this peculiar character are *L. dipterota*, *L. discreta, L. gradsteinii, L. lumbricoides, L. mizutanii, L. pectinella, L. stephaniana* and *L. utriculata*. Most species, however, lack such a large rectangular cell and sometimes the cell are of the same size as the first tooth as in *L. albescens*, *L. compressiuscula*, *L. javanensis*, *L. microloba, L. sordida* and *L. tjibodensis*.

5) Laminal cells

**Fig. 15:**
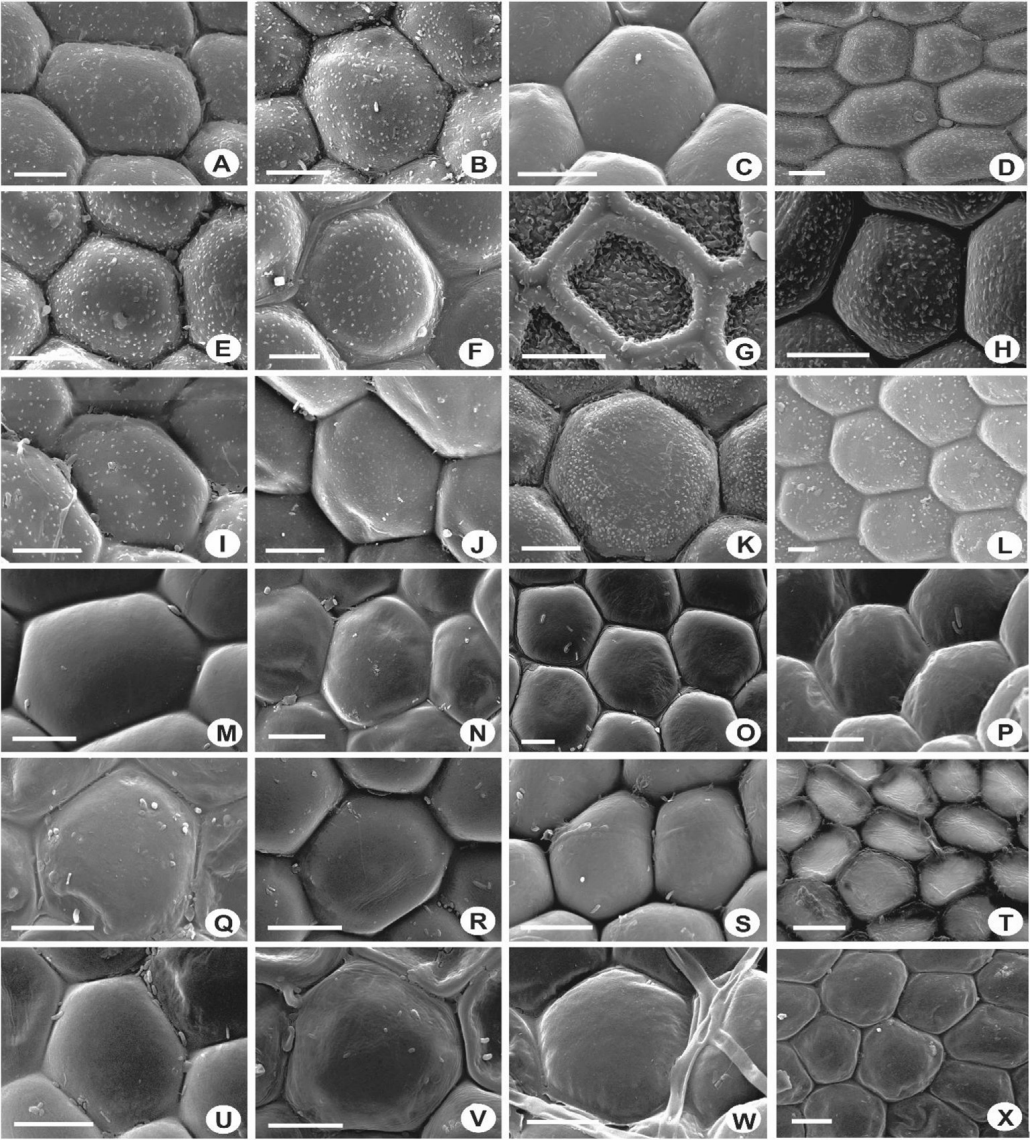
Laminal cells, showing rough cuticles with verrucose-small papillae (A-F, H-L), rough cuticles with tuberculate-small warts, coarser than papillae (G), and smooth cuticles (M–X). A: *L. pectinella*; B*: L. dipterota*; C: *L. pulchriflora*; D: *L. mimula*; E: *L. fleischeri*; F: *L. microloba*; G: *L. tuberculosa*; H: *L. discreta*; I: *L. sordida*; J: *L. utriculata*; K: *L. albescens*; L: *L. kinabalensis*; M: *L. micholitzii*; N: *L. alata*; O: *L. lumbricoides*; P: *L. exilis*; Q: *L.* papilionacea; R: *L. umbilicata*; S: *L. cocoes*; T: *L. stephaniana*; U: *L. dimorpha*; V: *Lejeunea* sp.; W: *L. eifrigii*; X: *L. patriciae*. Scale bar A–X: 10 μm.

The size of the cells of leaf lobes is usually rather uniform, gradually become smaller towards the leaf margin and larger towards the leaf base in most of the taxa. The cells are quadrate to rectangular at the margin, round to hexagonal in the middle, and tend to become rectangular to oblong towards the base. In a few species, the cells are distinctly differentiated, abruptly smaller at the middle of the leaf and becoming more or less elongated at the leaf base e.g., in *L. contracta* and *L. gradsteinii*. The cell walls are mostly hyaline to somewhat yellowish, thin to rather thick, and with or without trigones and intermediate thickenings. Normally there is only one intermediate thickening between adjacent trigones on the cell wall, but in *L. dimorpha, L. micholitzii*, *L. patriciae* and *L. pulchriflora,* 2(3) intermediate thickenings are present between adjacent trigones particularly at the base of the leaf. The surface of the cells or cuticle can be observed clearly through SEM in which superficially, three types of cuticle are seen i.e. smooth (Fig. 15M–X), verrucose-small papillae (Fig. 15A–F, H–L), and tuberculate-small warts, coarser than papillae (Fig. 15G). The latter, however, is proven to be cuticular wax projections [5,6].

6) Oil Bodies

**Fig. 16:**
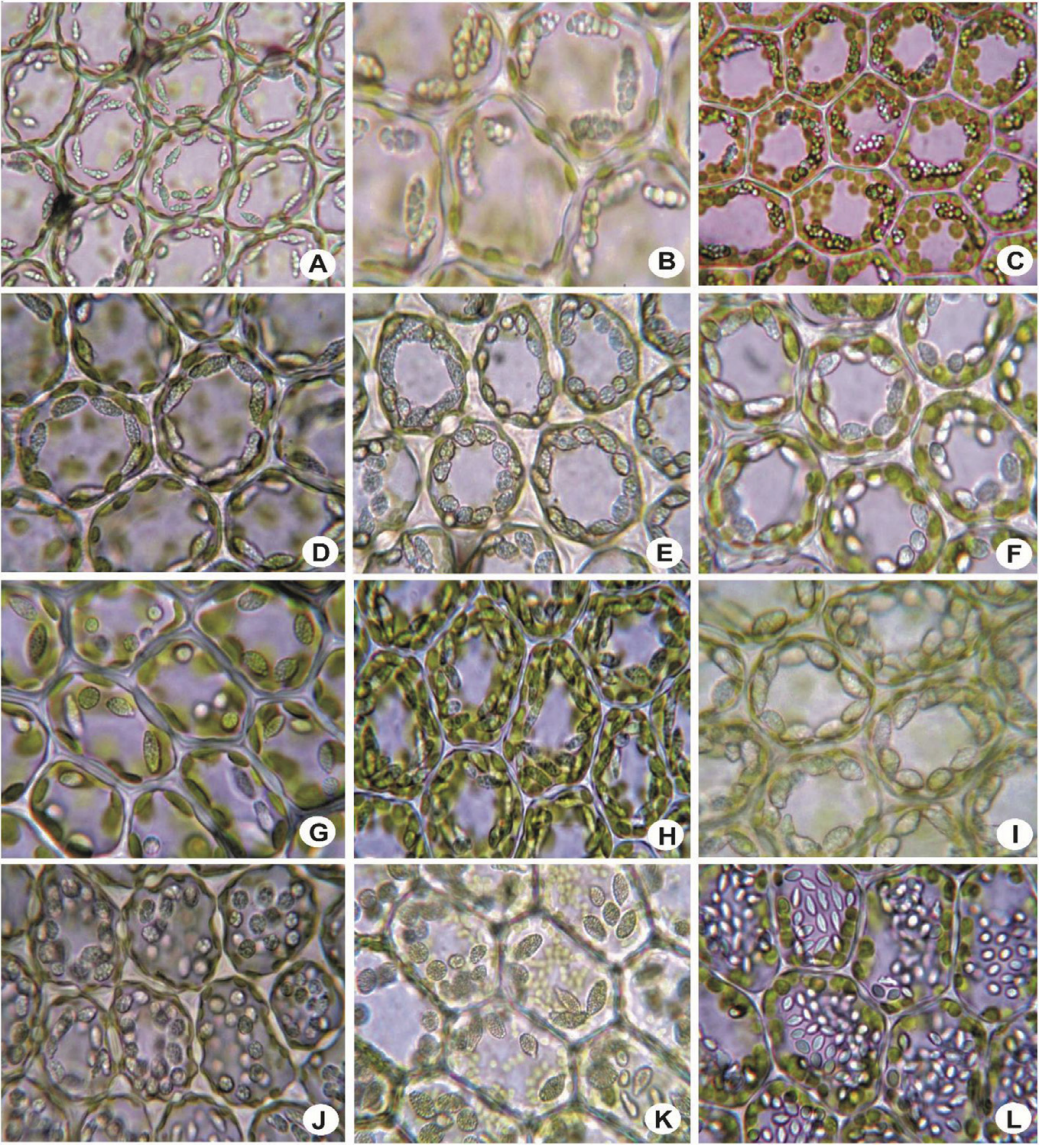
Oil bodies, showing glistering-botryoidal *Calypogeia*-type (A–C); somewhat glistening to faintly opaque-granular, *Jungermannia*-type (D–J); opaque-papillose, rough *Jungermannia*-type (K); glistening homogeneous, *Massula*-type (L). A: *L. albescens*; B: *L. sordida*; C: *L. anisophylla*; D: *L. mimula*; E: *L. umbilicata*; F: *L. discreta*; G: *L. tuberculosa*; H: *L. patriciae*; I: *L. kinabalensis*; J: *L. micholitzii*; K: *L. eifrigii*; L: *L. dimorpha*.

Four types of oil bodies of *Lejeunea* observed are given in Fig. 16 i.e. i) Glistening-botryoidal (*Calypogeia*-type); ii) Opaque-papillose (rough *Jungermannia*-type); iii) Somewhat glistening to faintly opaque-granular (*Jungermannia*type); and iv) Glistening-homogeneous (*Massula-*type). See Ref. [2] for the description of the oil bodies.

7) Gametoecia

Most of the *Lejeunea* studied are dioicous and 10 species are autoicous viz. *L. alata, L. anisophylla, L. compressiuscula, L. dimorpha, L. eifrigii, L. javanensis, L. flava, L. obscura, L. papilionacea* and *L. pulchriflora.*

**Fig. 17:**
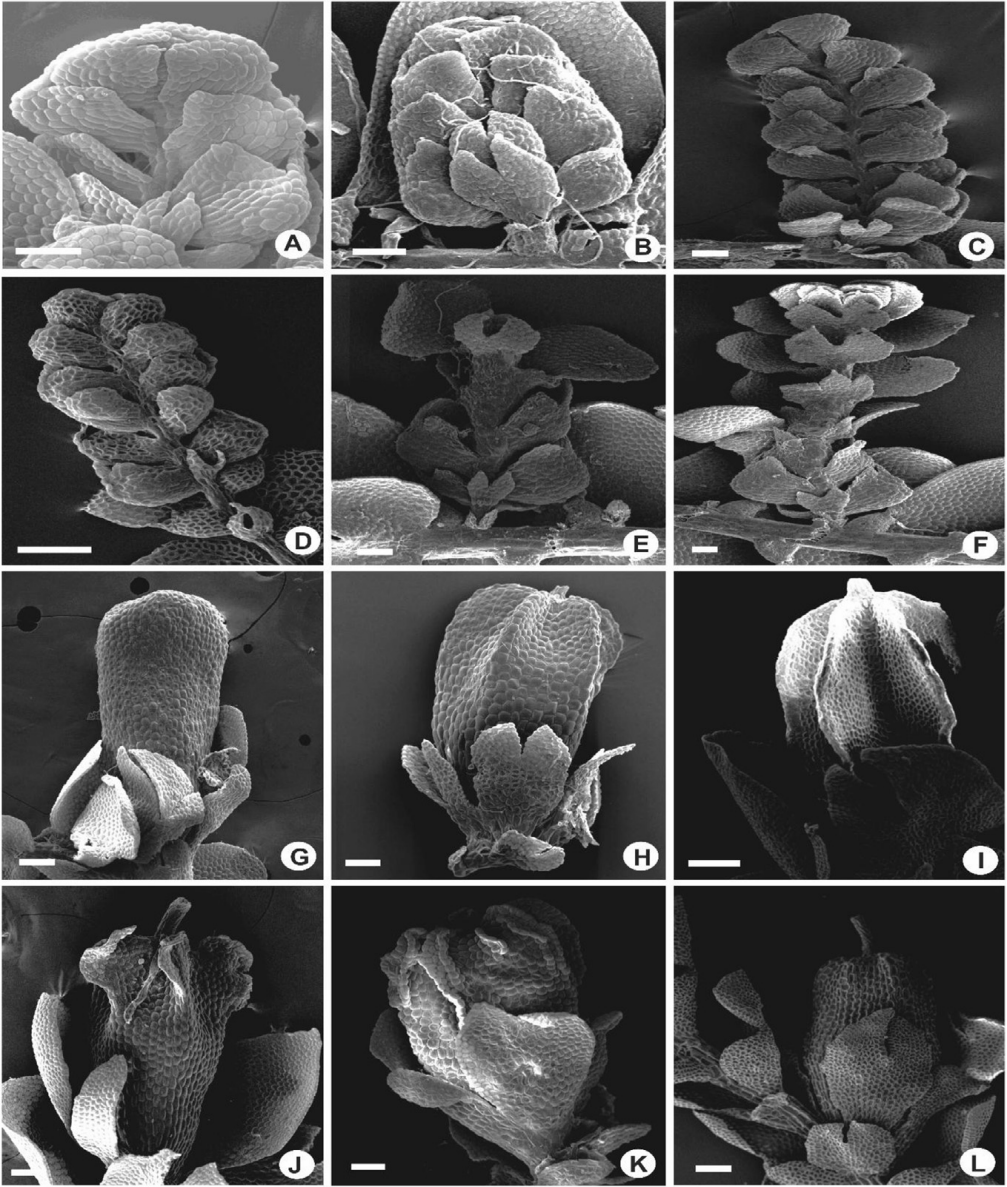
The position of androecial shoot. A–C: On long or short lateral branches; D: Terminal on main shoot; E–F: Intercalary on lateral branches. G–L: The morphology of perianth keels and beak. G: Eplicate, without keel and beak (*L. umbilicata*); H–I: Five keels with short-cylindrical beak (*L. kinabalensis, L. stephaniana*); J: Keels undulate and auriculate with long beak (*L. lumbricoides*); K: Keels 2-winged (*L. dipterota*); L: Five keels with long beak (*L. utriculata*). Scale bar A–L: 100 μm.
ii) Gynoecia: The gynoecia occur on short or long lateral branches and only sometimes on the main shoots. The arrangement pattern is usually monochasial in most of the *Lejeunea* observed, producing one innovation from each node of gynoecium but in *L. flava, L. fleischeri,* and *L. mimula,* dichasial arrangement is recorded in which two innovations are seen growing from the node of gynoecium. In most of the species, there is usually one gynoecium produced in a lateral position, however, at times, 2–5 gynoecia are arranged in a lateral row due to repeatedly fertile innovations e.g., in *L. alata*, *L. eifrigii, L. flava, L. lumbricoides* and *L. umbilicata*. The innovation of leaf sequence in all the *Lejeunea* in this study is lejeuneoid. The female bracts are loosely arranged to somewhat crowded, usually erect-spreading when moist and occasionally enveloping the perianth. They are 2-lobed and usually smaller than the normal leaves or almost of the same size. The bract lobes are usually ovate or obovate and are oblong and elliptic in a few species. The margins are entire in most of the species but strongly crenulate in *L. albescens, L. kinabalensis* and *L. pectinella*. The bract lobules are oblong or obovate and some are elliptic and lanceolate, 1/2–4/5 as long as the lobes in most species, but somewhat reduced in *L. eifrigii* and *L. alata*. The apex of bract lobule is acute, acuminate, obtuse or truncate. The bract lobules of *L. patriciae* sometimes bear marginal rhizoids at the apex. At the lateral side of the free margin, a special development of sinus with a hyaline papilla sometimes occurs, as in *L. utriculata* and in some populations of *L. patriciae* and *L. mimula*. The female bracteoles are ovate to obovate and usually longer than the underleaves or almost as long as the bract lobes. In *L. pectinella* and *L. mimula*, the bracteoles are longer than the bract lobes and the normal leaves. They are plane, seldom recurved and usually gradually tapering towards their bases but in *L. micholitzii, L. microloba, L. mimula, L. sordida, L. utriculata* and *L. wightii,* they abruptly taper towards their bases. The bracteole lobes are distant to overlapping and up to 1/3–2/3 of bracteole length. Perianths are obovoid in most of the species but clavate in *L. alata* and *L. eifrigii* and emergent up to 1/5–3/4 of perianth length when mature. The perianths are often with 5 prominent keels on the lateral and ventral side and occasionally weakly keeled at the dorsal side. However, perianths without keels (Fig. 17G) are also produced in a few species such as in *L. albescens, L. microloba* and *L. umbilicata*. The keels are usually mammillose and sometimes extended above more or less as auricles e.g., *L. lumbricoides* and *L. papilionacea* (Fig. 17J). The presence of this auricle is variable within the same species and is of little taxonomic significance. The keels are often winged in *L. dipterota, L. patersonii* and *L. pulchriflora*. The perianth apices are rounded to truncate and their beaks can be short, usually 2–3 cells high (e.g., *L. anisophylla*), or long with 6–10 cells high in *L. lumbricoides, L. mizutanii, L. pectinella* and *L. utriculata*. The beaks are short-cylindrical in most of species but trumpet-shaped in *L. discreta* and funnel-shaped in *L. mimula*. The cells of perianth are round, quadrate to subrectangular with well-developed or indistinct trigones and intermediate thickenings as those found on the leaf cells, and have a smooth to somewhat rough cuticle (Fig. 18). The rough cuticle on the perianths tend to become opaque e.g., *L. flava* and *L. tuberculosa.* The perianth characters are variable and rarely used as a diagnostic character in *Lejeunea*, however, in some cases the perianth characters are sufficient to separate some of the species e.g., *L. pectinella* and *L. discreta*.

i) Androecia: The androecial shoots occur mostly on short or long lateral branches and only occasionally terminal on branches or main shoots (Fig. 17A–F). The androecial shoot usually consists of 3–6 pairs of male bracts, sometimes up to 8–15 pairs, each enveloping 2 antheridia and 1–2(3) male bracteoles. The male bracts are closely imbricate, always smaller than the normal leaves, and with more strongly inflated and larger lobules. The lobules are hypostatic in which the free margin closely overlaps the younger bract and are somewhat smaller or almost the same size as the lobes. The apex of the lobule is usually obtuse to acute without the first tooth and occasionally with hyaline papilla e.g., *L. exilis*. Hyaline papilla is also seen in the uppermost lobe cell at insertion. The free margin is always flat, never recurved and the keel is strongly inflated and curved. The bract keels are entire or crenulate with wing or without wing. The male bracteoles are usually lobed but in *L. exilis*, they are sometimes unlobed, 4–5 cells long and 2 cells wide. The male bracteoles are somewhat similar to the normal underleaves but often slightly smaller (sometimes slightly larger as in *L. papilionacea*) and present only at the base of the androecial shoot. The presence of male bracteoles throughout the androecial shoot is not seen in the *Lejeunea* studied. Antheridia were seen in *L. albescens, L. exilis, L. lumbricoides, L. micholitzii, L. sordida* and *L. umbilicata,* usually with two antheridia per bract and with somewhat yellowish to hyaline stalk.

**Fig. 18:**
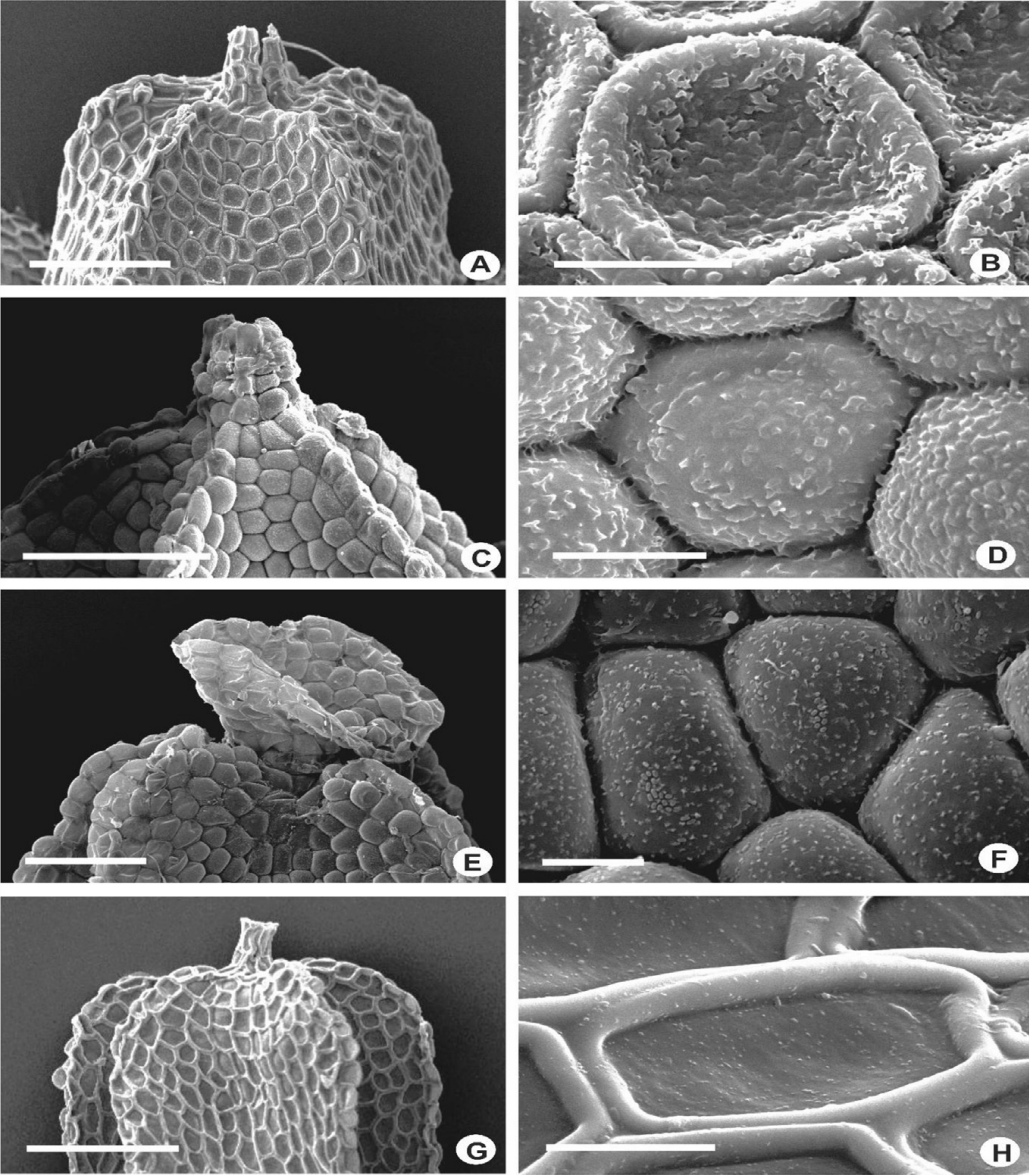
The perianth of *Lejeunea*, showing rough cells. A–B: *L. tuberculosa*; C–D: *L. flava*; E–F: *L. mimula*; G–H: *L. patersonii*. Scale bar A,C,E,G: 100 μm and B,D,F,H: 10 μm.
8) Sporophytes

**Fig. 19:**
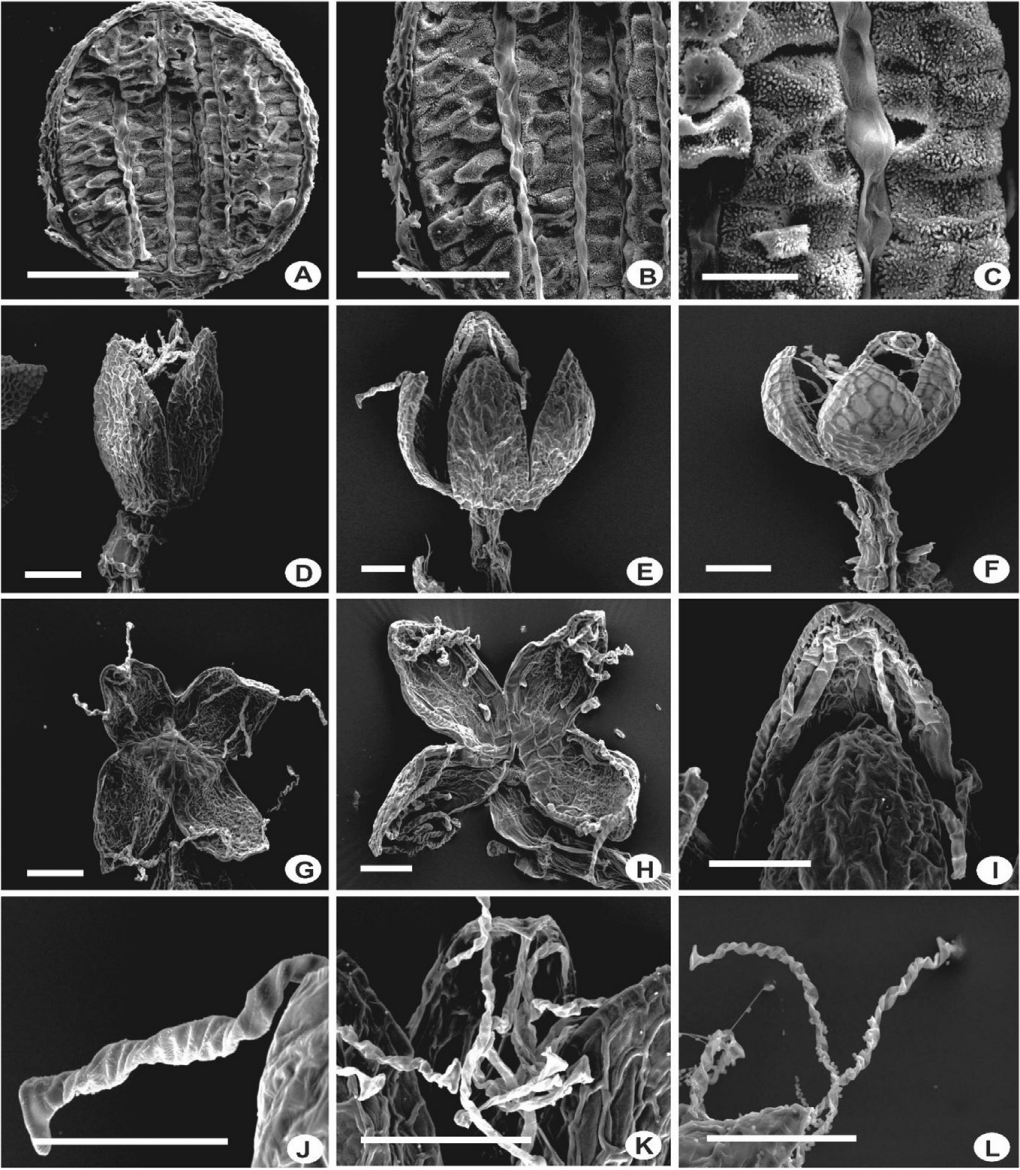
A–C: Longitudinal section of mature capsules (*L. flava*). D–F: Dehisced capsules. D: *L. flava*; E: *L. mimula*; F: *L. lumbricoides*; G–I: Inner valve surface. G: *L. flava*; H,I: *L. mimula*; J–L: Elaters with weak spiral bands of thickenings. J: *L. mimula*; K: *L. papilionacea*; L: *L. flava*. Scale bar A,B,D–L: 100 μm and C: 10 μm.

**Fig. 20:**
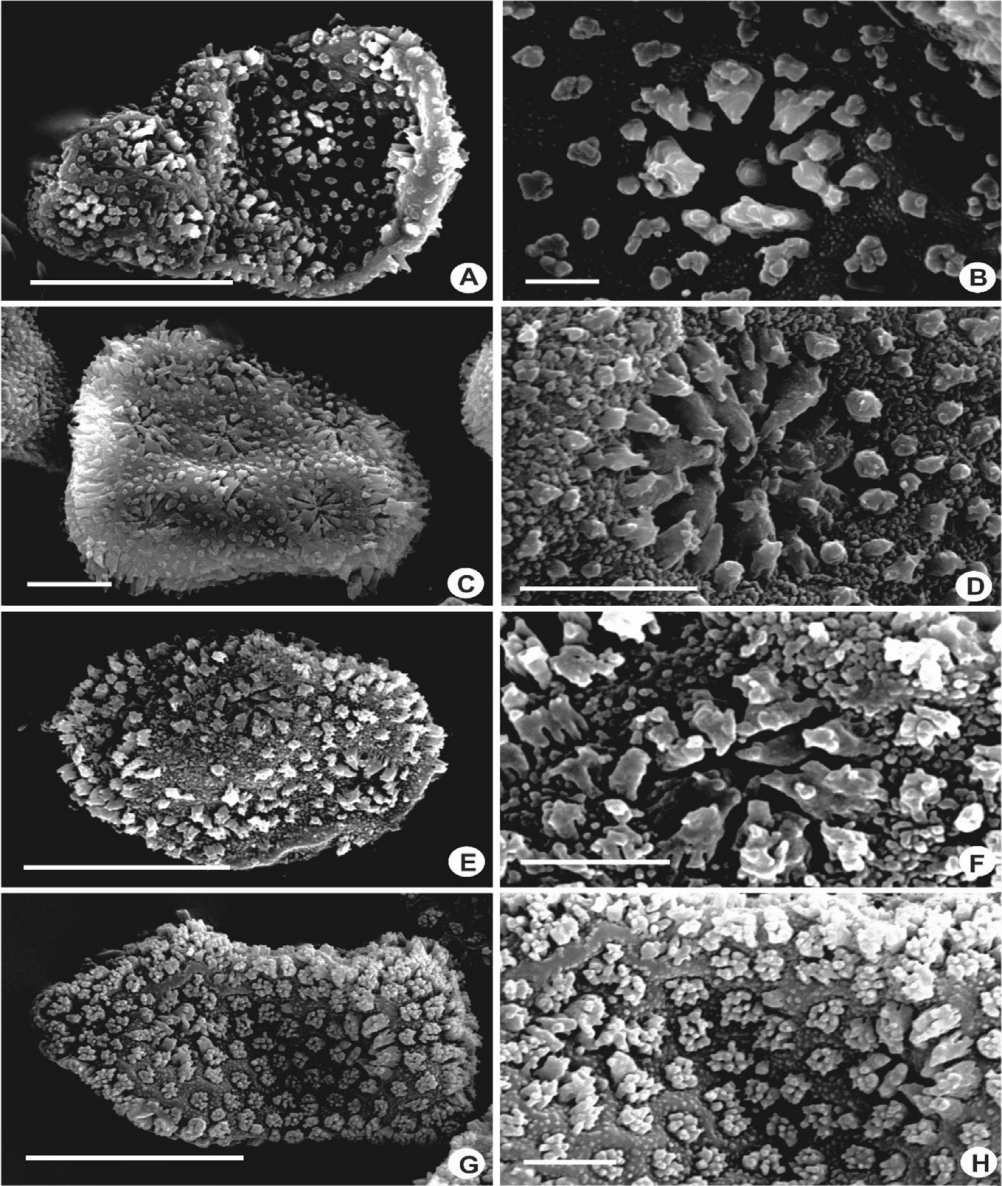
Mature spores, showing spore surface with rosettes and foramina. A–B: *L. mimula*; C–D: *L. flava*; E–F: *L. papilionacea*; G–H: *L. pulchriflora*. Scale bar A,C,E,G: 10 μm and B,D,F,H: 2 μm.

Generally, the sporophyte in *Lejeunea* has an articulate, hyaline seta with 12 outer cells and 4 inner cells in cross-section, nodular-type of capsule without fenestrate sheeth of thickening (Fig. 19). The elaters are faintly brown or hyaline with incrassate walls and weak spiral bands of thickenings (Fig. 19J–L). They are attached to the upper margin of each capsule-valve. The spores are arranged in decussate tetrads (Fig. 19A–C) and spore surfaces are with rosettes, spines and foramen (Fig. 20) Sporophytes were observed in nine species viz. *L. anisophylla, L. eifrigii, L. exilis, L. flava, L. lumbricoides, L. mimula, L. papilionacea, L. pulchriflora* and *L. tuberculosa*. Vegetative propagation is described in Refs. [2,7].

## 2 Experimental design, materials, and methods

All the specimens of *Lejeunea* including types were examined thoroughly in order to assess their morphological variation. Materials used for SEM study were the dried herbarium specimens and fresh specimens from the field. Specimens were first examined under a compound microscope to select suitable samples such as gametophytes, perianths and spores. The selected samples were soaked with 4% Glutaraldehyde for 12–24 hours at 4 °C. Then, the samples were washed with 0.1 M Sodium Cocodylate Buffer for three changes of 10 minutes each and followed by the dehydration process with ethanol series of 30%, 50%, 70%, 80%, 85%, 90%, 95% and three changes of 100% for 10 minutes in each period. The prepared material was then transferred into specimen basket and critical point dried in a critical point dryer (Thermo VG Scientific Polaran Critical Point Dryer 7501) for about 30 minutes. All the materials were then mounted directly on double-sided tape attached on top of aluminum stubs and coated with a thin layer of gold using a Polaran Sputter Coater. Specimens were viewed under the SEM microscope (Leo 1450 VPSEM) and photographs were taken for appropriate parts.
